# Mild Organosolv Delignification of Residual Aspen Bark after Extractives Isolation as a Step in Biorefinery Processing Schemes

**DOI:** 10.3390/molecules27103185

**Published:** 2022-05-17

**Authors:** Matiss Pals, Maris Lauberts, Douwe S. Zijlstra, Jevgenija Ponomarenko, Alexandr Arshanitsa, Peter J. Deuss

**Affiliations:** 1 Latvian State Institute of Wood Chemistry, 27 Dzerbenes Street, LV-1006 Riga, Latvia; marislauberts@gmail.com (M.L.); jevgenijaponomarenko@inbox.lv (J.P.); arshanica@edi.lv (A.A.); 2 Department of Chemical Engineering (ENTEG), University of Groningen, Nijenborgh 4, 9747 AG Groningen, The Netherlands; d.s.zijlstra@rug.nl (D.S.Z.); p.j.deuss@rug.nl (P.J.D.)

**Keywords:** aspen bark, lignin-first biorefining, organosolv lignin

## Abstract

European aspen (*Populus tremula* (L.) (*Salicaceae*)) bark is a promising raw material in multi-step biorefinery schemes due to its wide availability and higher content of secondary metabolites in comparison to stem wood biomass. The main objective of this study was to investigate the major cell wall component-enriched fractions that were obtained from aspen bark residue after extractives isolation, primarily focusing on integration of separated lignin fractions and cellulose-enriched bark residue into complex valorization pathways. The “lignin first” biorefinery approach was applied using mild organosolv delignification. The varying solvent systems and process conditions for optimal delignification of residual aspen bark biomass were studied using a response surface methodology approach. The conditions for maximum process desirability at which the highest amount of lignin-enriched fraction was separated were as follows: 20-h treatment time at 117 °C, butanol/water 4:1 (*v*/*v*) solvent system with solid to liquid ratio of 1 to 10. At optimal separation conditions, lignin-enriched fraction exhibited a higher content of β–O–4 linkages vs. C–C linkages content in its structure as well as a high amount of hydroxyl groups, being attractive for its further valorization. At the same time, the content of glucose in products of cellulose-enriched residue hydrolysis was 52.1%, increased from 10.3% in untreated aspen bark. This indicates that this fraction is a promising raw material for obtaining cellulose and fermentable glucose. These results show that mild organosolv delignification of extracted tree bark can be proposed as a novel biorefinery approach for isolation of renewable value-added products with various application potentials.

## 1. Introduction

European aspen is popular species common in European temperate climate zones. In Latvia, European aspen growth uses 10% of the area of all forests [1]. Aspen tree is widely used in production of cellulose, chips, plywood, pellets, etc. [2]. In most of these processes debarking takes place, and together with targeted products this produces a considerable amount of waste in terms of discarded tree bark. This makes wood bark, and more precisely aspen bark, a widely available lignocellulosic feedstock [3]. For example, the forestry sector in Latvia produces around 400,000 m^3^ of wood bark waste annually [1]. Most of the produced bark is used as a fuel. Bark as a forestry sector waste product is produced year-round in relatively steady quantities in timber processing plants. However, periods of low heat demand combined with issues related to long-term storage of wood bark in piles outdoors, it is combusted for destruction without any economic gain [4]. This leads to wood bark being a considerably cheaper lignocellulosic feedstock than for example wood chips or other available resources [5]. Wood bark is a lignocellulosic resource with a wide range of possible applications. There have been multiple studies beyond the energy utilization of bark as lignocellulosic resource with focus on extractives isolation [6]. Bark has been extensively studied as a source of biologically active extractives suitable for various pharmaceutical and cosmetological applications [7,8,9]. For example, due to the high content of salicylates, aspen bark water-soluble extracts are commercialized as cosmetics components with skin conditioning and antimicrobial properties [10]. However, relatively little work has been done to establish biorefinery pathways of holistic bark processing incorporating extractives removal and further valorization of obtained residue by separating its main components: lignin, cellulose, and monomeric carbohydrates [11,12,13].

**Figure 1:**
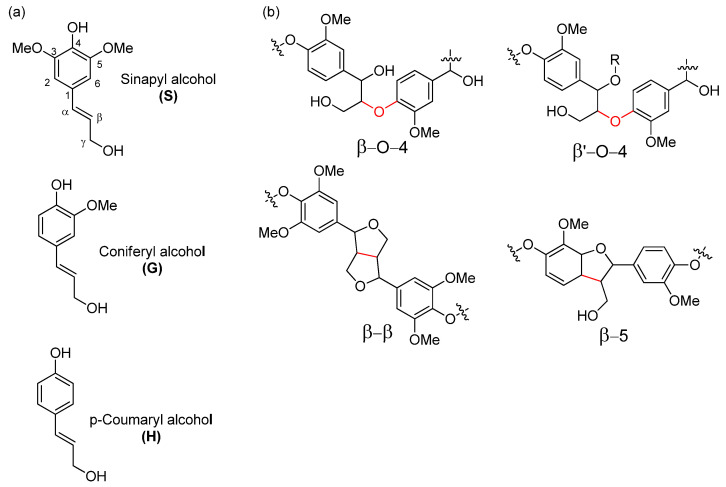
(**a**) Structure of the most common monolignols. (**b**) The most commonly occurring lignin linkage motifs.

As a renewable aromatic biopolymer making from 15 to 35% of the bark mass, lignin holds the key for maximizing value extraction from bark biomass. In tree bark lignin content is noticeably higher than in wood, since the bark is the barrier that protects the tree from biotic and abiotic factors. Furthermore, lignin gives the necessary structural strength and together with non-lignin phenolics provides anti-microbial protection [14,15,16]. Lignin valorization is currently hindered by the application of delignification methods that significantly alter the native lignin structure to give highly recalcitrant materials [17,18]. In hardwood such as European aspen, lignin is a complex macromolecule that mostly originates from p-coumaryl alcohol, coniferyl alcohol, and sinapyl alcohol through radical polymerization, providing a polymer made up of H, G and S units, respectively (Figure 1a) [19].

The most abundant lignin linkage motif that the radical polymerization generates is the β–O–4 motif, which can make up to 70% of all linkage motives (Figure 1b). In addition, lignin also contains native C–C linkage motifs such as β–5, 5–5, β–β. In contrast to the β–O–4 motif, these latter bonds are much more resistant to chemical alteration and generally remain unaltered during pretreatment and fractionation processes.

Lignin in hardwoods such as aspen has more S subunits and less C–C bonds, making it easier to separate than softwood species such as pine [20,21,22,23]. High-quality lignin in context of its valorization is defined as lignin with abundant aryl−ether linkages, including β–O–4 linkages, for its application in synthesis of versatile polymers as macromonomer [24,25] or as a source of renewable monomeric phenolics instead of fossil-based raw materials. These low molecular weight compounds can be used as building block chemicals in different polycondensation reactions, which are versatile in the polymer industry [26].

A promising approach for obtaining high-quality lignin from lignocellulosic biomass as residual bark is mild organosolv delignification. Relatively little has been done in terms of tree bark organosolv delignification, and to our knowledge European aspen bark organosolv delignification has not been previously studied. Organosolv been described in literature for quite a long time now. Organosolv delignification is a biomass fractionation method using organic solvents, often in the presence of acidic catalyst, that allows efficient fractionation of biomass in its major components (cellulose, lignin, and hemicellulose in soluble monosaccharide fraction) at relatively mild conditions, with temperatures between 100 °C to 200 °C and pressure from atmospheric to 20 bar. In comparison with kraft and sulphite delignification, the organosolv approach is a more environmentally friendly process due to lack of harmful sulphur-containing emissions.

In a typical organosolv delignification process, lignin undergoes partial acid catalyzed depolymerization that can undergo recondensation, resulting in condensed oligomeric lignin fragments with relatively low molecular mass. However, the structural transformation of lignin is highly dependent on the separation conditions. Mild organosolv at temperatures below 120 °C has the advantage that it leaves the more regular β–O–4 intact, and by using specific solvents and extraction setups similar extraction efficiencies can be obtained. Recent work at moderate temperatures (~100–150 °C) and using organic acids or low concentration of mineral acids allows us to obtain relatively low condensed lignin with a high amount of native β-O-4 bonds and good solubility in organic solvents [27,28]. Furthermore, extraction efficiency can be further enhanced using flow-through setups that lower the exposure time of released fragments to the reactive medium [29,30]. Use of organic solvents in separation allows us to increase the efficiency of the process when compared to dilute acid hydrolysis methods due to the increase of lignin solubility in the used process medium. In addition, the use of alcohols in the organosolv process promotes incorporation of ethers chains into the α position of the phenyl propane units. This structure is called β‘-O-4 and is shown in Figure 1b. This incorporation protects the native lignin β-O-4 structure from degradation and re-condensation, allowing separation of low condensed lignin more suitable as a feedstock for further valorization pathways while also increasing lignin solubility in organic solvents that can be useful for further processing. [5,17,27,31,32,33,34]. Therefore, mild organosolv delignification of residual European aspen bark using low molecular weight alcohol solvent systems can be a promising approach for fractionation of this underutilized resource to obtain fractions suitable for further use as starting materials in biorefinery processing clusters.

**Figure 2:**
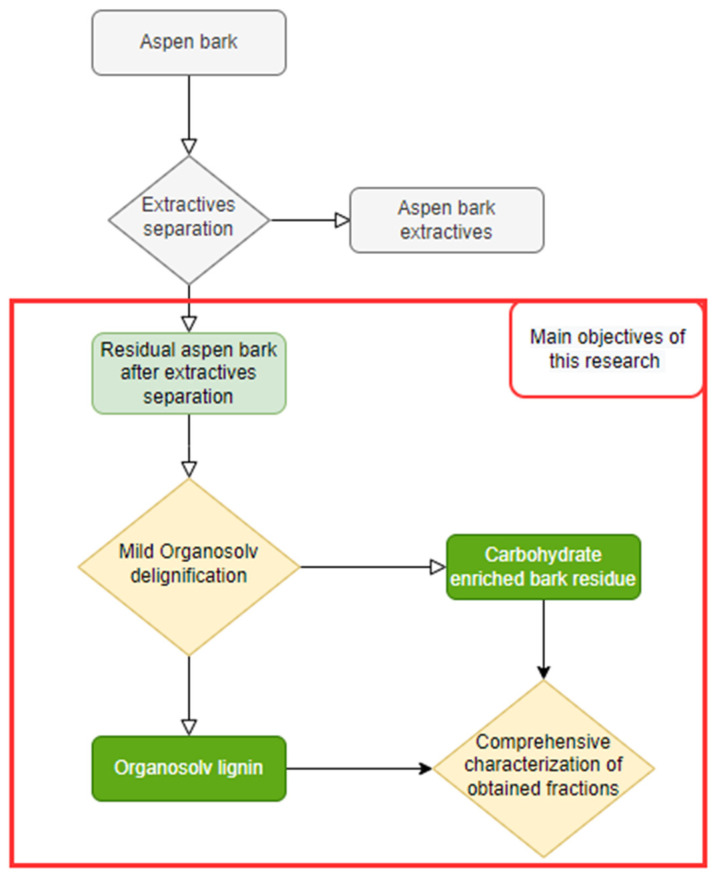
Process diagram of the workflow for the biorefinery approach for valorization of aspen bark.

The scope of this research is to study the process of mild organosolv delignification of extracted aspen using two components mixes of protic and aprotic organic solvents with alcohols and water in terms of the yield and characteristics of precipitated lignin and non-soluble cellulose enriched fraction, to show that used process is suitable for successful aspen bark valorization in biorefinery processing scheme. Process overview is shown in Figure 2. 

## 2. Results

### 2.1. Isolation of Extractives

Extractives including lipophilic, non-lignin phenolic, and carbohydrates compounds were separated from aspen bark utilizing the accelerated solvent extraction system (ASE). Extraction conditions were selected based on previous work done in our laboratory to maximize the yield of extractives [6,35]. To separate polar, semi-polar and non-polar secondary metabolites, sequential extraction was performed. The first extraction solvent was n-hexane, followed by ethyl acetate, and finally a 40% ethanol water solution (*v*/*v*). Yields of the separated extractives increased with the increase of the polarity of the utilized extraction solvents. n-hexane soluble extractives made up 5.9 ± 0.7% (wt %) of the biomass, the ethyl acetate fraction 8.0 ± 0.6% and the ethanol soluble fraction 11.9 ± 0.7% of the biomass. The total yield of separated extractives from aspen bark was 25.9 ± 0.9%. Chemical composition and possible directions of practical applications of separated extracts is under development by our group, and since it is not a focal point of this publication it will not be discussed here. 

A substantial (70–80%) part of the biomass is left after these steps, of which the components need to be effectively separated for efficient utilization in integrated biorefinery scheme development.

### 2.2. Characterization of Residual Aspen Bark after Extractives Isolation

The composition of bark before and after extraction was characterized using analytical pyrolysis (Py-GC/MS) by categorizing volatile degradation products formed from biomass components during their thermal decomposition in conditions of analytical pyrolysis. The diagnostic volatile pyrolysis products were classified as:

- Carbohydrates-derived compounds represented mainly by aliphatic acids and esters, aliphatic alcohols, aliphatic aldehydes and ketones, furan and pyran derivatives, cyclopentane derivatives, anhydro sugars;
- Non-methoxylated aromatic compounds derived mainly from phenolic extractives;
- Methoxylated phenols, guaiacyl (G-) and syringyl (S-) derivatives derived from lignin;
- Lipophilic extractives-derived compounds (Table 1).

The full list of identified diagnostic compounds is shown in supplementary information.

According to Py-GC/MS data the extracted bark has significantly lower content of hydrophilic (phenolic) and lipophilic extractives in comparison to the untreated bark (as relative content of corresponding volatiles decreased from 14.90 to 2.15%). The content of lipophilic extractives-derived compounds also decreased from 5.69 to 1.94%. Thus, overall, hydrophilic extractives were efficiently isolated from bark, while a significant part of lipophilic extractives remained in the bark biomass. The relative summary content of lignin-derived G- and S-compounds in untreated aspen bark was 18.18%, while after extraction it was 24.85%. The relative content of carbohydrate-derived compounds in initial bark and extracted residue was 61.23% and 71.06%, correspondingly. So, the isolation of extractives leads to enrichment of both lignin and carbohydrates content in bark biomass, enhancing its potential as feedstock for obtaining these compounds.

### 2.3. Residual Aspen Bark Organosolv Delignification

**Figure 3:**
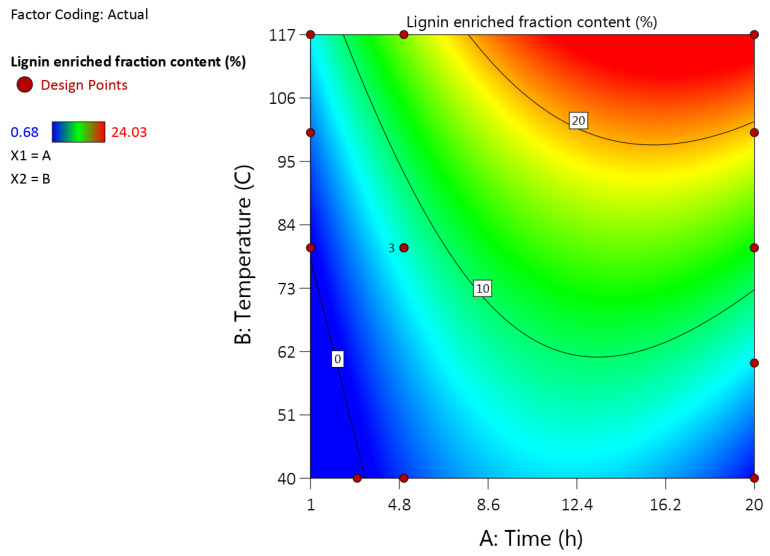
The gravimetric isolated yield of precipitated lignin-enriched fractions in dependence of temperature and process time.

To select the optimal solvent system for aspen bark delignification, several options were tested. Delignification conditions remained constant for this set of experiments: 5 h process duration at a temperature of 80 °C and a solid to liquid ratio of 1 to 10. These conditions were selected using previous experience as a starting point for used solvent system optimization [36]. In total, five different solvent systems were tested. Obtained results in terms of lignin-enriched fraction yield obtained from bark as well as obtained residual fraction yield are shown in Figure 3. 

It was shown that the higher yield was obtained using the 1,4-dioxane/ethanol and n-butanol/water solvent mixtures. For further experiments an n-butanol/water solvent system was being selected as optimal since adequate results can be achieved and butanol is a green solvent. The next aim was to optimize the delignification conditions. In total, four time durations at five different temperatures were tested (1, 3, 5, 20 h and 40, 60, 80, 100, 117 °C (refluxing)). The results of twenty experiments differing by delignification regimes in terms of lignin- and cellulose-enriched fractions yield are presented in Figure 3 and Table 2. Input data is shown in supplementary information (Table S2).

Both temperature and duration of processing enhance the yield of lignin-enriched fractions. The highest yield of 24.03% on DM of extracted biomass was achieved at a temperature of 117 °C and 20 h of duration. 

**Figure 4:**
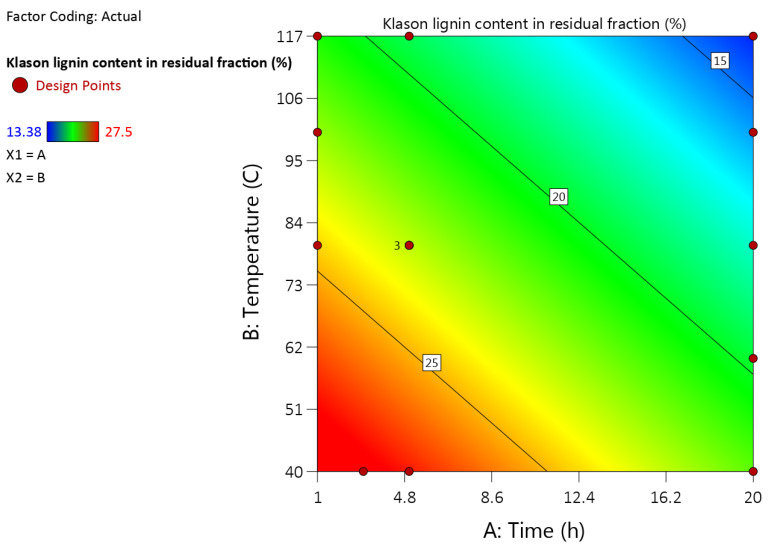
Klason lignin content in residual fraction in dependence of temperature and process time.

It was shown that increasing temperature and duration of mild organosolv treatment highly promotes the dissolution of biomass components in the n-butanol-water solution, decreasing the yield of the insoluble cellulose-enriched fraction from 90% to 43% while simultaneously increasing the purity of the separated cellulose-enriched fraction in terms of leftover lignin admixtures (Figure 4).

**Figure 5:**
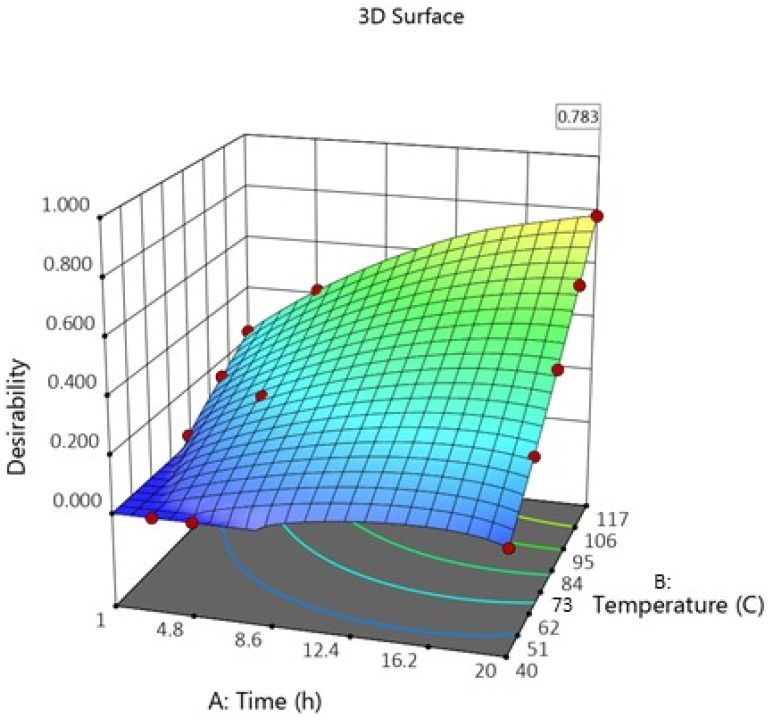
Desirability plot of produced model in terms of time and temperature of delignification reaction.

Using the obtained data, a full factor experimental model was created using DesignExpert 13 software. The following goals were set for the model: maximize the obtained lignin-enriched fraction yield with minimum admixture content while simultaneously obtaining maximum amount of residual cellulose-enriched fraction. The calculations take all these factors into consideration and provide desirability scores for all model variables. The obtained desirability plot for this model is shown in Figure 5. 

The obtained model shows that a more favorable outcome of delignification reaction can be obtained when utilizing conditions with longer treatment time and higher temperature. At the boiling point of butanol (117 °C) and treatment time of 20 h, model desirability was 0.783. From this, these conditions were chosen as most suitable for residual aspen bark delignification. It is important to mention that although higher temperatures do increase the lignin-enriched fraction yield, it can also promote degradation of lignin. Due to this, it vital to perform complete characterization of obtained lignin fractions. 

### 2.4. Characterization of Lignin-Enriched Fraction

#### 2.4.1. Composition of Lignin-Enriched Fraction Isolated from Bark by Solvents of Different Polarity

To compare different solvent systems used in lignin separation, it is vital to perform comprehensive characterization of obtained lignins in terms of admixture content and obtained lignin structure. It was shown that the content of admixtures in precipitated fractions is significantly dependent on the polarity of solvent used for delignification. 

**Figure 6:**
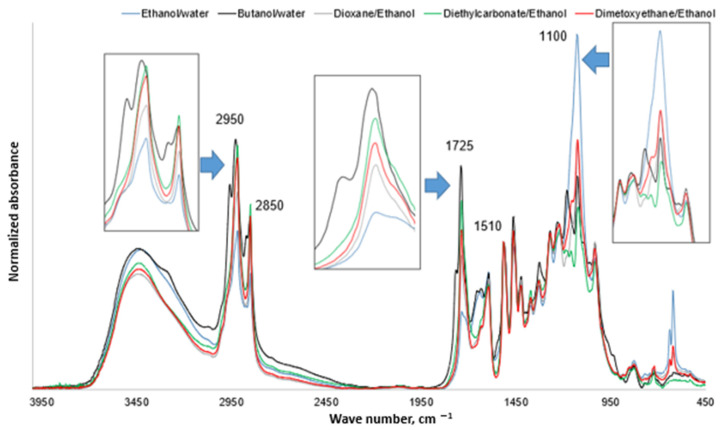
FTIR spectra of obtained lignin-enriched fractions normalized at 1510 cm^−1^.

The FTIR spectra (Figure 6) of the lignin-enriched fraction showed that the fraction isolated by the most polar ethanol/water solvent contains the least amount of lipophilic admixtures that could originate from fatty acids—glycerol and hydroxyl fatty acid-ferulic acid esters of suberin. This was evidenced by the least absorbance intensity at 2950–2850 cm^−1^ attributed to the C-H stretch in methyl and methylene groups as well as the least absorbance at about ~1725 cm^−1^ attributed to the presence of unconjugated carbonyls in ester groups. However, the most absorbance intensity in the region of 1100 cm^−1^ attributed to the C–O deformation in secondary alcohols and aliphatic ethers indicates that this fraction contains the most of carbohydrates admixtures. In contrast, the fraction isolated by the least polar solvent (diethyl carbonate/ethanol) contained the least amount of carbohydrates admixtures but a significantly higher amount of lipophilic compounds in comparison with fraction isolated by polar ethanol–water solvent. For all solvents except n-butanol-water with decreasing solvent polarity, the content of lipophilic admixtures in precipitated fraction is decreased, increasing the content of carbohydrates admixtures. This dependence was not observed for n-butanol-water solvent. It highly promotes the solubility of lipophilic compounds of bark that were co-precipitated with lignin where water was added as a co-solvent.

The analysis of carbohydrate admixtures after performing hydrolysis of obtained lignin fractions and determining carbohydrates by GC-FID method showed that the only carbohydrates present in lignin-enriched fractions were xylose and arabinose, with content less than 4%. Analytical pyrolysis showed that when using the n-butanol/water solvent system, the obtained lignin-enriched fraction contains a significant (20.8 ± 0.4%) amount of carbohydrate admixtures, which are not detected using GC-FID alditol carbohydrate method of analysis. This can be explained by the formation of hydrolysis-stable n-butanol-carbohydrate ethers during the delignification process, as described in the literature [30].

The application of analytical pyrolysis allowed obtain additional information concerning composition of lignin enriched fractions. This method is widely used for the characterization of the chemical composition of lignocellulosic biomass by the determination of pyrolysis products referring to the definite groups of biomass substituents [37]. The volatile pyrolysis products were classified as carbohydrates-derived volatiles, lignin-derived volatiles (consisting of compounds described in Section 2.2), nitrogen-containing volatiles and aliphatic long-chain and cyclic compounds derived from lipophilic extractives. Data from Py-GC-MS (Table 3) reveals that dominant non-phenolic admixtures present in the lignin-enriched fraction are lipophilic compounds most likely coming from suberinics present in hardwood tree bark. The lowest amount of lipophilic admixtures was found in the fraction obtained using the n-butanol/water system, reaching 26.4%.

When comparing the FTIR spectra of the lignin-enriched fraction obtained by n-butanol/water mild organosolv delignification with widely available and pure LignoBoost lignin obtained from hardwood biomass, the presence of lipophilic admixtures is clearly confirmed with an increase of peaks at 2950 cm^−1^ and 1725 cm^−1^. The amount of lipophilic admixtures in the lignin-enriched fraction is an important factor that needs to be further studied for effective utilization of lignin-enriched fractions, both in terms of admixture effect on lignin fraction usability and possibilities of admixture separation. 

#### 2.4.2. Structural Characterization of Lignin-Enriched Fraction

The structure of lignin constituents of separated lignin-enriched fractions, including the distribution of monolignols and dominant linkages between them as determined by semi-quantitative 2D HSQC NMR analysis, are shown in Table 4.

**Figure 7:**
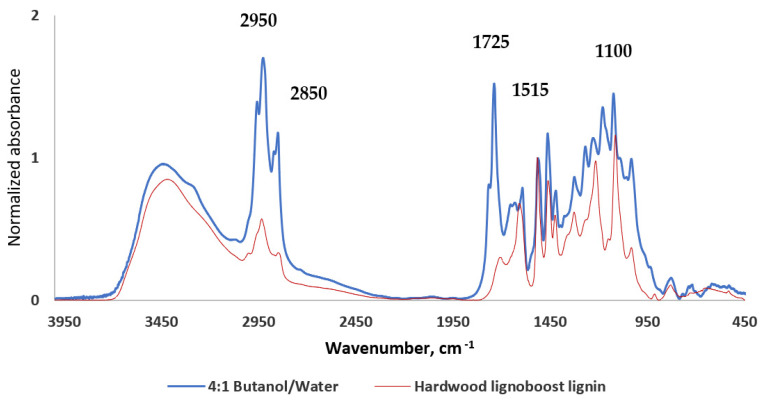
FTIR spectra of lignin-enriched fraction obtained with butanol/water solvent system compared to hardwood LignoBoost lignin, both normalized at 1515 cm^−1^.

From obtained data (Figure 7), it can be seen that by varying used solvent systems, monolignol distribution remains similar, with the exception of the diethyl carbonate/ethanol mixture that promotes lignin condensation and new C–C bond formation of S lignin subunits (Table 4). In terms of linkage unit content per structural subunit of lignin, the highest amount of β–O–4 and β’–O–4 linkages were obtained by performing delignification with n-butanol- and 1,4-dioxane-containing solvent systems. 

From samples under study, the fractions isolated with the diethyl carbonate/ethanol solvents system revealed themselves as most condensed due to lowest content of remaining β–O–4 linkages and appearance of S condensed structures non-typical for native lignin (Table 4). Among others, lignins isolated by n-butanol/water and ethanol/water solvent systems are most enriched with β–O–4 linkages and therefore can be characterized as the least condensed in comparison with other samples, including commercial hardwood LignoBoost lignin. Moreover, the high content of β’–O–4 structures in isolated lignins, which are absent in commercial lignin, can be estimated as a favorable factor that allowed avoiding undesirable condensation of lignin structures in the pretreatment stages of its chemical modification [38].

When lignin fractions were obtained at 80 °C and at 117 °C (Table 5), it was noticeable that an increase in temperature did not dramatically increase unwanted condensation of obtained lignin fractions, indicated by the high number of β-O-4 groups present in lignin that was obtained in harsher conditions. 

Obtained lignin-enriched fractions are suitable for further utilization as feedstock for obtaining polymeric materials, including polyurethane and polyepoxides, since they are enriched with reactive -OH groups (both aliphatic and phenolic), as determined with ^31^P NMR spectroscopy (data shown in Table 6). In the fractions obtained, the portion of aliphatic groups most reactive in reactions of nucleophilic attachment consisted of 65–81% from total OH content. This can be recognized as a significant advantage of isolated lignins versus commercial lignins, including Alcell, Indulin AT, Sarkanda, Curan-2711P in which the aliphatic groups portion was lower and varied in the range of 27.6–54.2% [39].

For lignin that was obtained using a n-butanol/water solvent system, at harsher conditions (20 h, 117 °C), aliphatic –OH group content was 2.20 (mmol·g^−1^) and total phenolic—OH group content was 0.83 (mmol·g^−1^). This indicates that while an increase in temperature does not lead to loss of lignin β–O–4 bonds, it does leads to loss of aliphatic hydroxyl groups to some extent. Nonetheless, obtained lignins, even at harsher conditions, are still enriched with reactive –OH groups and can be successfully used as a natural polymeric feedstock in further valorization. The example of ^31^P NMR spectra of Butanol/Water organosolv lignin is shown in Supplementary Materials (Figure S2).

### 2.5. Characterization of Carbohydrate-Enriched Fraction

The yield of carbohydrates-enriched fraction obtained as residue after delignification varied between 63.6% and 82.4%. 

Carbohydrate-enriched fraction was characterized in terms of lignin admixture content by determining Klason lignin content in obtained samples. Obtained results are shown in Table 7. 

When comparing lignin amounts in residuals with starting feedstock it is notable that obtained residues are considerably cleaner than lignin admixtures. With varying solvents Klason lignin content in residuals ranged from 25.5% to 14.9% and a butanol/water solvent system obtained a residual fraction that contained 15.7% of lignin admixtures. By increasing delignification condition harshness, at 117 °C and in a 20 h timeframe, it was possible to obtain purer residual fractions containing only 13.66% lignin admixtures. When considering individual carbohydrate content in obtained residual fractions, obtained results were similar with all used solvent systems. All samples contained predominantly glucose (Glc) with some admixtures of xylose (Xyl) and arabinose (Ara) (Table 5).

Total carbohydrate content in these samples varied between 39.8% to 51.2%, and when using butanol as a delignification solvent, total carbohydrate content was 42.8%. When performing delignification at harsher conditions with n-butanol as a solvent at 117 °C for 20 h, the residual fraction obtained at this regime was more enriched with carbohydrates and contained the least amount of lignin admixture. Total carbohydrates content in this sample was 61.23%, with a glucose portion of 85%, indicating that this obtained fraction is enriched with cellulose and is promising feedstock in further applications. 

## 3. Materials and Methods

### 3.1. Sample Preparation

European aspen (*Populus tremula*) bark samples were collected from 30 year-old trees grown in the Talsu region in Latvia. Bark was manually removed and air dried until a moisture content of 10–15% was reached and then ground to smaller particle size (<2 mm) using a Retch-100 cutting mill. Prior to use, the milled bark was stored in plastic bags in a freezer at −18 °C. 

### 3.2. Extractives Isolation Using ASE

Bark samples were extracted using a Dionex ASE 350 accelerated solvent extraction system (Thermo Scientific, Waltham, MA, USA) with sequential extraction by solvents of increasing polarity—hexane, ethyl acetate followed by 40% EtOH solution in water (*v*/*v*). The cumulative yield of extractives isolated by sequential extraction was ~35% The details of extraction are described elsewhere [6]. The extracted bark was oven-dried at 50 °C and stored in desiccator with pentoxide as a desiccant.

### 3.3. Organosolv Delignification

Organosolv delignification of extracted aspen bark was performed by the method described by [29], which focused on the isolation of lignin from walnut shell. About 10 g of extracted aspen bark was weighed in a round-bottom flask and 100 mL of the used solvent system (Ethanol/Water, N-butanol/Water, 1,4-Dioxane/Ethanol, Diethyl carbonate/Ethanol, Dimethoxymethane/Ethanol) was added together with 1.65 mL of HCl (35%, *w*/*w*) as a catalyst. The reaction mixture was heated at varying temperatures (40, 60, 80, 100, 117 °C) and process durations (1, 3, 5, 20 h). Afterwards, the reaction mixture was filtered and the separated cellulose-enriched fraction was washed with acetone (3 × 25 mL), air-dried, oven-dried in a vacuum with paraffin as solvent adsorbent, and weighed. Azeotrope butanol-water mix was evaporated and re-dissolved in 2–5 mL of acetone. Afterwards, this mixture was precipitated in 300 mL of cold deionized water. After 24 h, precipitated lignin-enriched fraction was filtered through a glass frit filter (P2), dried in vacuum oven, and weighed. The yield of biomass remaining in soluble state in water-organic solutions was calculated by difference of substrate weight before delignification and summing weight of separated lignin-enriched and cellulose-enriched fractions. The water solution remaining after filtration of lignin-enriched fractions was lyophilized and the solid obtained was used for analysis. 

### 3.4. Wet Chemistry Analysis

Ash content in biomass was measured as a residue after ignition at 550 ± 1 °C in a Carbolite ELF 11/6B furnace. 

The content of acid-insoluble lignin (Klason lignin) in extracted bark and cellulose-enriched residue was determined by TAPPI Standard method, T 222 om-98. Correction was performed on a dry ash free basis.

Total monomeric carbohydrate content in starting feedstock and residual pulp were determined by the gas chromatography with flame ionization detection (GC-FID) method, as described elsewhere, with slight modifications [40]. Thus, 10 mg of the sample was loaded into a screw tube, 0.125 mL of 72% H_2_SO_4_ was added and the mixture was incubated at room temperature for 3 h. Then, 3.5 mL of deionized water was added and the mixture was incubated at 101 °C for 3 h. The cooled sample was neutralized with 0.32 mL of NH_4_OH and 0.1 mL of methyl-α-D-glucopyranoside solution (20 mg·mL^−1^) was added as an internal standard. Then, 0.2 mL of the sample was taken and 1 mL of 2% NaBH_4_ solution in dimethyl sulfoxide was added, and the mixture was incubated at 40 °C for 90 min. The excess of NaBH_4_ was decomposed by adding 0.1 mL of glacial acetic acid, and 2 mL of acetic anhydride and 0.2 mL of 1-methylimidazole were added to the solution and incubated at 40 °C for 10 min. Then, 5 mL of water was added, and the solution was extracted with 1 mL of CH_2_Cl_2_ for 1 min. The CH_2_Cl_2_ layer was collected for GC analysis. Analysis was performed using an Agilent 6850 series GC instrument with 30 m DB-1701 column.

### 3.5. Heteronuclear Single Quantum Coherence (HSQC) Spectroscopy

Structural analysis of lignin-enriched fractions was performed by HSQC NMR spectroscopy. For analysis, 60 mg of lignin-enriched fraction was dissolved in 0.7 mL of d6-acetone. A few drops of D2O were added to ensure complete solubility of the lignin. The NMR analysis was performed on a Bruker Ascend™ Neo 600 using the following parameters: (F2 = 11 to −1 ppm), (F1 = 160 to −10 ppm), nt = 4, ni = 512, d1 = 1.5, CNST [2] = 145, and pulse sequence hsqcetgpsi2. Analysis was performed with MestReNova. The obtained values for the detectable linking motifs (β–O–4, β’–O–4, β–β, and β–5) were divided by a factor of 1.3 as the HSQC measurements overestimate these values, as shown elsewhere [36].

### 3.6. ^31^P NMR Spectroscopy

The content of hydroxyl groups was determined by using methodology based on work conducted by Dimitris Argyropoulos et al. [41]. Thus, 40 mg of lignin-enriched fraction was dissolved in a 1.6 mL of a mixture of pyridine and CDCl_3_ (1.6:1, *v*/*v*). To prepared sample solution, 200 μL of internal standard solution (9 mg·mL^−1^ N-Hydroxy-5-norbornene-2,3-dicarboxylic acid imide, in pyridine and CDCl_3_ 1.6:1 *v*/*v*) and 50 μL of a Cr(acac)_3_ solution (5.6 mg·mL^−1^ in pyridine and CDCl_3_ 1.6:1 *v*/*v*) were added. After thorough stirring, 100 μL of of Cl-TMDP was added, and the sample was stirred at room temperature before being transferred to an NMR tube and measured.

### 3.7. Analytical Pyrolysis (Py-GC-MS)

Analytical pyrolysis was performed using a Frontier Lab Micro-shot Pyrolizer Py-2020iD (temperature of pyrolysis 773 K, heating rate 600 K s^−1^), directly coupled with a Shimadzu GC/MS-QP 2010 apparatus, with a capillary column RTX-1701 (injector temperature, 250 °C, MS scan range m/z 15–350 Da, carrier gas helium). The mass of a sample was 1.00–1.10 mg. The oven program was 1 min of isothermal at 333 K, then heating at 6 K min^−1^ to 543 K and 10 min storage. The identification of the individual compounds was performed on the basis of GC/MS data using the Library MS NIST 147.L113, whereas the relative area of the peaks of individual compounds was calculated by using Shimadzu software on the basis of GC/FID data. Three repeated tests were performed, and averaged data were used for discussion, with the standard deviation for the relative area of individual components being less than 5.0%.

### 3.8. Data Processing

A full-factor experimental plan was performed in addition to data visualization using DesignExpert 13 (Version number 13.0.5.0 ×64). Necessary calculations were performed using Microsoft Excel (Version number 16.0.15028.20160). 

## 4. Conclusions

Obtained results indicate that European aspen bark is a promising feedstock for a multi-step biorefinery approach. After separation of non-lignin phenolic extractives, obtained residual bark is a valuable resource for obtaining lignin and carbohydrates due to the concentration of these components in residual bark. Residual aspen bark can be successfully separated into major components utilizing a mild organosolv delignification approach with an n-butanol/water solvent system. Best results in terms of lignin-enriched fraction yield were achieved by delignification performed during 20 h at a temperature of 117 °C. At these conditions, separated lignin-enriched fractions exhibited a high amount of β–O–4 linkages as well as a high amount of hydroxyl groups, indicating a high viability of separated lignin-enriched fractions for its further valorization as a macromonomer for polymer materials. At optimal conditions, obtained cellulose-enriched residue consisted of 52.1% glucose after hydrolysis, indicating that this fraction is a promising raw material for obtaining cellulose and fermentable glucose. 

## Figures and Tables

**Table 1:** Composition of diagnostic volatile pyrolysis products of untreated and extracted aspen bark.

Detected Diagnostic Pyrolysis Products	Untreated Aspen Bark	Aspen Bark after Isolation of Extractives
	Summary Peaks Area Normalized on Diagnostic Compounds, %	
Carbohydrates-derived compounds	61.23	71.06
Non-methoxylated aromatic compounds	14.90	2.15
Guaiacyl derivates	11.85	13.18
Syringyl derivates	6.33	11.67
Lipophilic extractives-derived compounds	5.69	1.94

**Table 2:** Precipitated lignin-enriched fraction and insoluble cellulose-enriched fractions yields on dry matter of extracted bark depending on used solvent system.

Solvent System *	ε (Dielectric Constant)	Lignin Enriched Fraction, %	Residual Fraction, %
ethanol/water	53.9	3.6	82.4
n-butanol/water	57.5	13.2	73.7
1,4-dioxane/ethanol	9.8	15.9	63.7
diethyl carbonate/ethanol	12.0	1.6	68.1
dimethoxy-ethane/ethanol	13.8	13.2	70.0

* Process conditions: 5 h, 80 °C, solid to liquid ratio 1:10, fraction yields calculated on dry matter of extracted bark.

**Table 3:** The content of the major groups of biomass constituents-derived products in diagnostic volatiles of precipitated lignin-enriched fractions, according to Py-GC/MS data, normalized to 100%.

	Content, %				
Solvent System	Identified	Carbohydrates	Lignin	N-Containing	Aliphatic
ethanol/water	97.9 ± 0.8	2.7 ± 0.2	56.7 ± 0.8	0.03 ± 0.2	38.5 ± 0.6
n-butanol/water	97.3 ± 0.3	20.8 ± 0.4	45.9 ± 0.9	0.48 ± 0.1	26.3 ± 0.4
1,4-dioxane/ethanol	99.6 ± 0.7	3.4 ± 0.1	45.9 ± 0.6	n.d.	50.1 ± 0.3
diethyl carbonate/ethanol	94.2 ± 0.8	3.4 ± 0.1	34.8 ± 0.8	n.d.	55.9 ± 0.9
dimethoxy-ethane/ethanol	98.1 ± 0.2	3.4 ± 0.2	46.1 ± 0.3	n.d.	48.6 ± 0.9

**Table 4:** Lignin-enriched fraction monolignols and dominant linkage distribution as determined by semi-quantitative 2D HSQC NMR analysis.

	HQSC NMR, Molar Ratio, (Per 100 Aromatic Units)							
	Monomeric Units			Linkages				
Solvent System	G	S	S *_con._	β–O–4	β’–O–4	β–β	β–5	Total Linkages
hardwood lignoBoost lignin	40	49	-	18	-	16	10	44
ethanol/water	43	57	-	24	41	12	5	82
n-butanol/water	44	55	-	21	46	11	4	82
1,4-dioxane/ethanol	45	55	-	14	51	11	3	79
diethyl carbonate/ethanol	36	55	9	3	35	9	9	58
dimethoxy-ethane/ethanol	44	56	-	15	35	11	9	72

* Condensed syringyl units.

**Table 5:** Lignin-enriched fraction, obtained using varied conditions, monolignols and dominant linkage distribution as determined by semi quantitative 2D HSQC NMR analysis.

	HQSC NMR, Molar Ratio, (Per 100 Aromatic Units)							
	Monomeric Units			Linkages				
Used Delignification Condition	G	S	S * _con._	β–O–4	β’–O–4	β–β	β–5	Total Linkages
Comercial hardwood lignoBoost lignin	40	49	-	18	-	16	10	44
5 h, 80 °C, solid to liquid ratio 1:10	44	55	-	21	46	11	4	82
20 h, 117 °C, solid to liquid ratio 1:10	45	55	-	10	51	10	-	71

* Condensed syringyl units.

**Table 6:** Hydroxyl group content in precipitated lignin enriched fractions.

	^31^P NMR–OH Group Content, (mmol·g^−1^) *					
Solvent System	Aliph.	S Unit.	G Unit.	H Unit.	COOH	Total
ethanol/water	4.01	0.13	1.25	0.22	0.04	5.65
n-butanol/water	3.90	0.09	0.72	0.02	0.07	4.81
1,4-dioxane/ethanol	4.23	0.35	0.96	0.04	0.04	5.62
diethyl carbonate/ethanol	3.27	0.05	0.81	0.01	0.03	4.17
dimethoxy-ethane/ethanol	3.32	0.43	0.91	0.06	0.03	4.76

* Analysis done in duplicate, with relative standard deviation being less than 10%.

**Table 7:** The yield of cellulose-enriched residues and their composition in terms of Klason lignin content and completely hydrolyzed carbohydrate content.

			Carbohydrates GC-FID, %							
Solvent System	Yield, %	KL Content, %	Rha	Fuc	Ara	Xyl	Man	Gal	Glc	Total
unextracted aspen bark	-	27.50	0.45	0.47	4.84	9.51	0.93	1.43	10.34	27.97
ethanol/water	82.40	25.50	0.50	n.d *	1.02	15.96	0.55	0.90	15.91	34.84
n-butanol/water	73.67	15.70	0.68	n.d	2.68	12.22	0.70	0.88	25.70	42.87
1,4-dioxane/ethanol	63.65	14.90	0.00	n.d	2.04	15.39	1.01	0.43	29.18	48.05
diethyl carbonate/ethanol	68.08	17.10	0.72	n.d	4.92	11.57	0.83	1.50	31.53	51.23
dimethoxy-ethane/ethanol	70.02	16.20	0.00	n.d	3.32	12.40	0.54	0.92	26.27	43.46

* n.d—not detected. Analysis done in duplicate, with relative standard deviation being less than 5%.

## Data Availability

Not applicable.

## Supplementary Materials

The following supporting information can be downloaded at: https://www.mdpi.com/article/10.3390/molecules27103185/s1, Figure S1: 2D HSQC NMR spectra of lignin enriched fraction obtained with butanol/water solvent.; Figure S2: P31 NMR spectra of lignin enriched fraction obtained with butanol/water solvent.; Table S1: Composition of diagnostic volatile pyrolysis products of untreated and extracted aspen bark Table S2: Input data for delignification model optimization.
