# Supplementary material for: Mild Organosolv Delignification of Residual Aspen Bark after Extractives Isolation as a Step in Biorefinery Processing Schemes
Source: Molecules. 2022 May 17;27(10):3185. doi: 10.3390/molecules27103185 (PMC9145397; doi:10.3390/molecules27103185)

Table S1. Composition of diagnostic volatile pyrolysis products of untreated and extracted aspen bark

Detected diagnostic pyrolysis products	Untreated aspen bark	Aspen bark after isolation of extractives
	Summary peaks area normalized on diagnostic compounds, %	
**Carbohydrates derived compounds**	**61.23**	**71.06**
Acetic acid, formic acid methyl ester, 2-oxo-propanoic acid, propanoic acid, 2-propenoic acid methyl ester, acetic acid methyl ester, 2-oxo-propanoic acid methyl ester, 2-hydroxyethyl 2-propenoic acid, ester, crotonic acid vinyl ester, propanoic acid, 2-methylpropyl ester, 2-methylene cyclopentanol, 2-propyl-1-heptanol, methylglyoxal, 2,3-butanedione, 1-hydroxy-2-butanone, hydroxy-acetaldehyde, 2,3-pentanedione, 1-hydroxy-2-propanone, hexanal, 1-hydroxy-2-butanone isomer, propanal, butanedial, 2,2-dimethyl-3-hexanone, 1-(acetyloxy)-2-propanone, 3-methyl 2-heptanone, 1-(acetyloxy)-2-butanone, (E)-2-pentenal, 4-heptenal, 4-methyl-2,3-pentanedione, pentanal, pentanadial, 2-cyclopenten-1-one, 2-methyl-2-cyclopenten-1-one, 4-cyclopentene-1,3-dione, 2,3-dimethyl-1,2-cyclopentanedione, 2-cyclopenten-1-one, 3-methyl-2-cyclopenten-1-one, 3-methyl-1,2-cyclopentanedione, 2-hydroxy-3-methyl-2-cyclopenten-1-one, 3-ethyl-2-cyclopenten-1-one, 3-ethyl-2-hydroxy-2-cyclopenten-1-one, 2-methyl-furan, 3-methyl-furan, 2,3-dihydro-3-methyl-furan, 2(3H)-furanone, 3(2H)-furanone, furfura, 2-furanmethanol, 5-methyl-2(5H)-furanone, acetylfuran, dihydro-4-hydroxy-2(3H)-furanone, 5-methyl-2-furancarboxaldehyde, dihydro-2(3H)-furanone, 2(5H)-furanone, 3-methyl-2,5-furandione, 3-methyl-2,5-furandione, 2,5-dihydro-3,5-dimethyl 2-furanone, 3-hydroxydihydro-2(3H)-furanone, 4-metyl-5H-furan-2-one, 5-(hydroxymethyl)dihydro-2(3H)-furanone, 5-acetyldihydro-2(3H)-furanone, 3-methyl-2,4(3H,5H)-furandione, 2,3-dihydro-benzofuran, 5-(hydroxymethyl)-2-furancarboxaldehyde, 5-hydroxymethyldihydrofuran-2-one, 3-hydroxy-,5,6-dihydro-(4H)-pyran-4-one, 3-hydroxy-2-methyl-4H-pyran-4-one, 3,5-dihydroxy-2-methyl-4H-pyran-4-one, dihydro-2H-pyran-3(4H)-one, 2-hydroxymethyl-5-hydroxy-2,3-dihydro-(4H)-pyran-4-one, 1,4;3,6-aianhydro-.alpha.-d-glucopyranose, D-(+)-ribonic acid .gamma.-lactone, 1,4;3,6-dianhydro-.alpha.-d-glucopyranose, 1,6-anhydro-.beta.-D-glucopyranose		
**Non-methoxylated aromatic compounds**	**14.90**	**2.15**
1-Ethenyl-2-methyl-benzene, 2-hydroxy-benzaldehyde, benzoic acid methyl ester, benzyl alcohol, phenol, 2-methyl-phenol, 2,3- dimethyl-phenol, 3-and 4-methyl-phenol, 4-methoxy-3-methyl-phenol, 3,4-dimethyl-phenol, 4-ethyl-phenol, 3',5'-dihydroxyacetophenone, benzoic acid, 2,6-dimethyl-phenol, 2-ethyl-4-methyl-phenol, 3-phenyl- 2-propen-1-ol (cinnamyl alcohol), 3-methoxy-5-methyl-phenol, 4-(2-propenyl)-phenol, 1,4-benzenediol, 2-methyl-1,4-benzenediol, 3-methoxy-1,2-benzenediol, 3-(p-hydroxyphenyl)-1-propanol.		
**Guaiacyl derivates**	**11.85**	**13.18**
Guaiacol, p-methylguaiacol, p-ethylguaiacol, p-vinylguaiacol, eugenol, trans-isoeugenol, cis-isoeugenol, vanillin, acetoguaiacon, guaiacylacetone, propioguaiacone, trans-coniferyl alcohol		
**Syringyl derivates**	**6.33**	**11.67**
Syringol, 4-methyl-syringol, 4-ethyl-syringol, 4-vinyl-syringol , 4-allyl- and 4-propyl-syringol, 4-propenyl-(cis)syringol, 4-propenyl-(trans)syringol, syringaldehyde, acetosyringone, syringylacetone.		
**Lipophilic extractives derived compounds**	**5.69**	**1.94**
Dodecane, 2,3-dihydro-1,4-dioxin, 1-nonene, 1,2-cyclohexanedione, 1-dodecene, tridecane, 1-tridecene, (Z)-2-pentene, 1-ethoxy-4-methyl, octanoic acid, tetradecane, (Z)-3-tetradecene, pentadecane, 1-pentadecene, 1,2-cyclohexanediol, tetradecane, 1-tetradecene, 1-pentadecene, 2-methyl-hexadecane, eicosane, 9-hexadecenoic acid, eicosane, isomer, (E)-3-eicosene, 8-hexyl-pentadecane, 2-methyl-eicosane, (E)-9-eicosene, 1-tricosene		

Dodecane, 2,3-dihydro-1,4-dioxin, 1-nonene, 1,2-cyclohexanedione, 1-dodecene, tridecane, 1-tridecene, (Z)-2-pentene, 1-ethoxy-4-methyl, octanoic acid, tetradecane, (Z)-3-tetradecene, pentadecane, 1-pentadecene, 1,2-cyclohexanediol, tetradecane, 1-tetradecene, 1-pentadecene, 2-methyl-hexadecane, eicosane, 9-hexadecenoic acid, eicosane, isomer, (E)-3-eicosene, 8-hexyl-pentadecane, 2-methyl-eicosane, (E)-9-eicosene, 1-tricosene

---

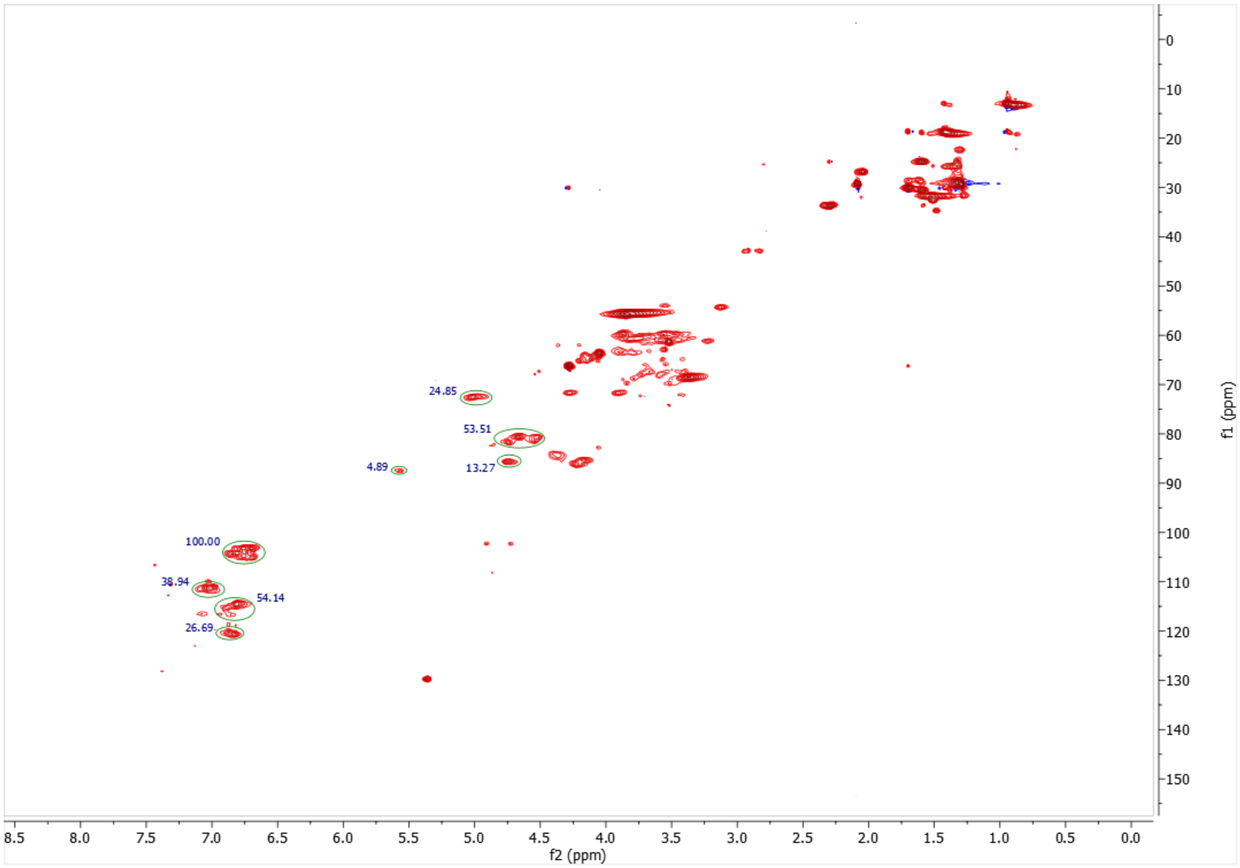

Figure S1. 2D HSQC NMR spectra of lignin enriched fraction obtained with butanol/water solvent.

Table S2. Input data for delignification model optimisation

Exp. Nr	Time	Temperature	Lignin yield, %	Residuals yield, %	Klason lignin in residuals %
1	1	40	0.36	97.15	28.05
2	3	40	0.68	98.29	27.50
3	5	40	0.75	99.53	25.26
4	20	40	1.54	92.77	13.84
5	1	60	0.49	98.54	25.03
6	3	60	1.83	93.46	25.70
7	5	60	2.50	90.65	23.98
8	20	60	5.18	84.79	22.46
9	1	80	2.16	93.08	24.54
10	3	80	4.57	87.38	24.10
11	5	80	6.76	94.13	20.66
12	20	80	11.88	63.48	18.40
13	1	100	3.23	81.78	25.15
14	3	100	11.37	73.06	19.45
15	5	100	11.87	63.84	17.78
16	20	100	21.75	43.75	13.38
17	1	117	4.34	81.44	23.73
18	3	117	12.87	71.07	19.84
19	5	117	18.38	61.55	16.09
20	20	117	24.03	43.51	13.66
21	20	117	23.81	41.60	12.16
22	20	117	22.73	40.52	12.59
23	20	117	25.00	42.11	14.64
24	1	80	2.87	91.67	22.77
25	1	80	2.32	92.07	23.77
26	1	80	2.96	91.70	25.77

Figure S2. P31 NMR spectra of lignin enriched fraction obtained with butanol/water solvent.

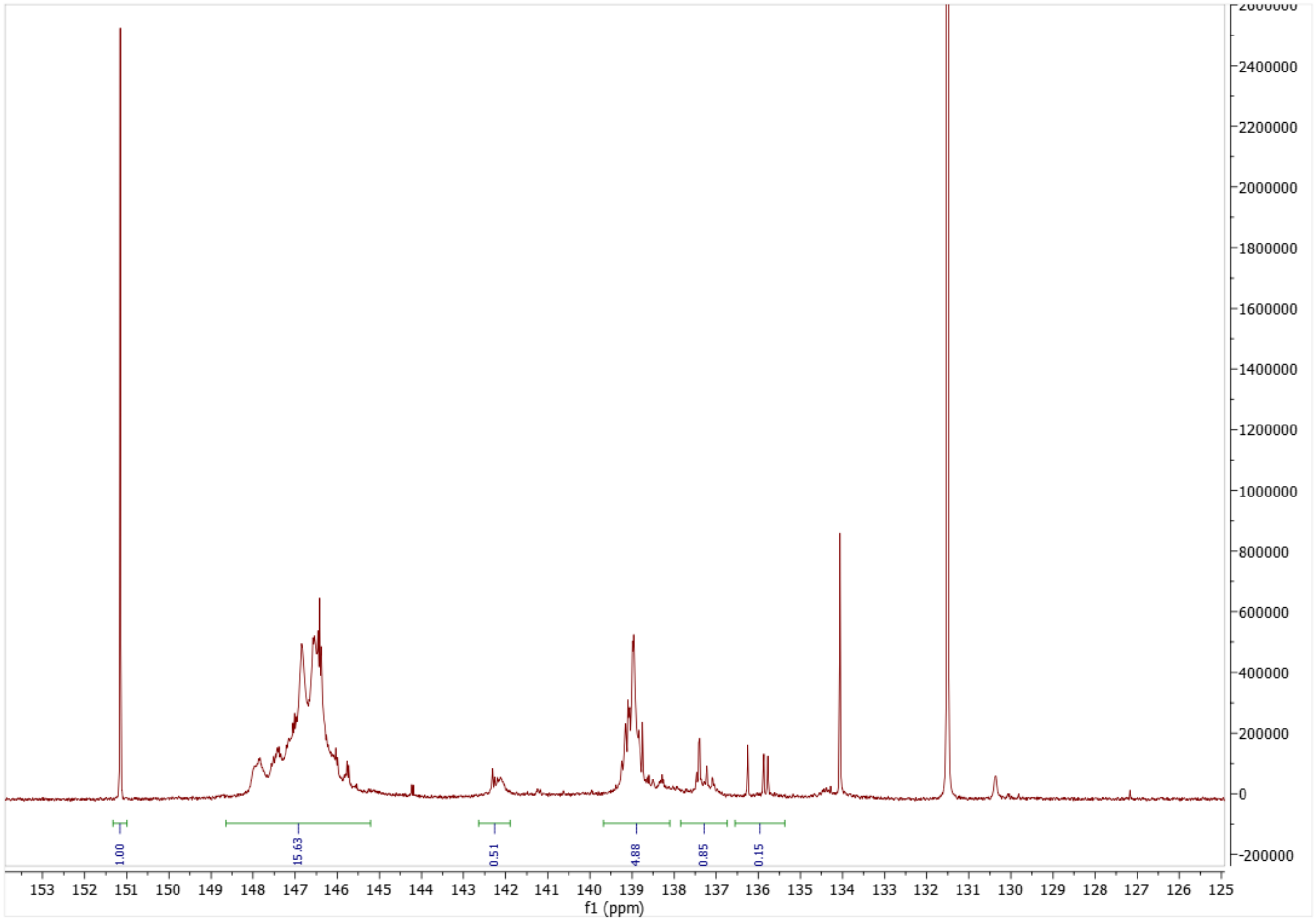

Supplement: Supplementary file 1 [file molecules-27-03185-s001.zip › molecules-1713633-supplementary.pdf]
